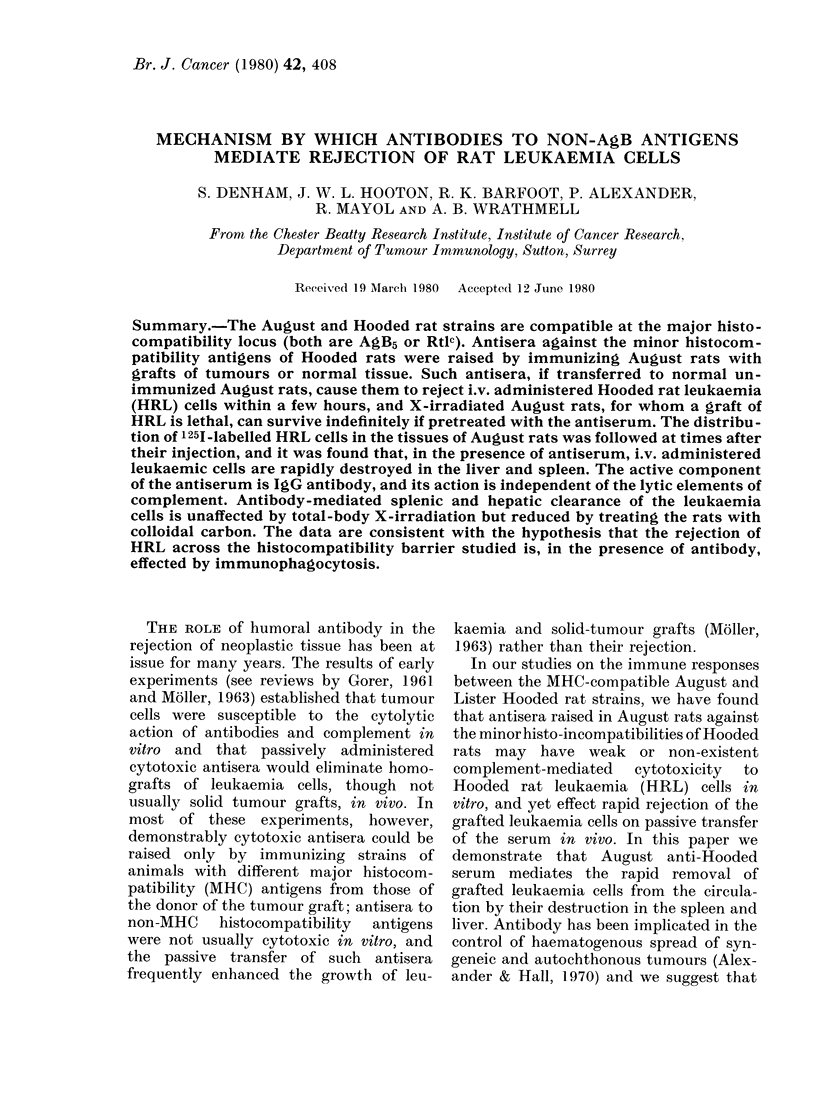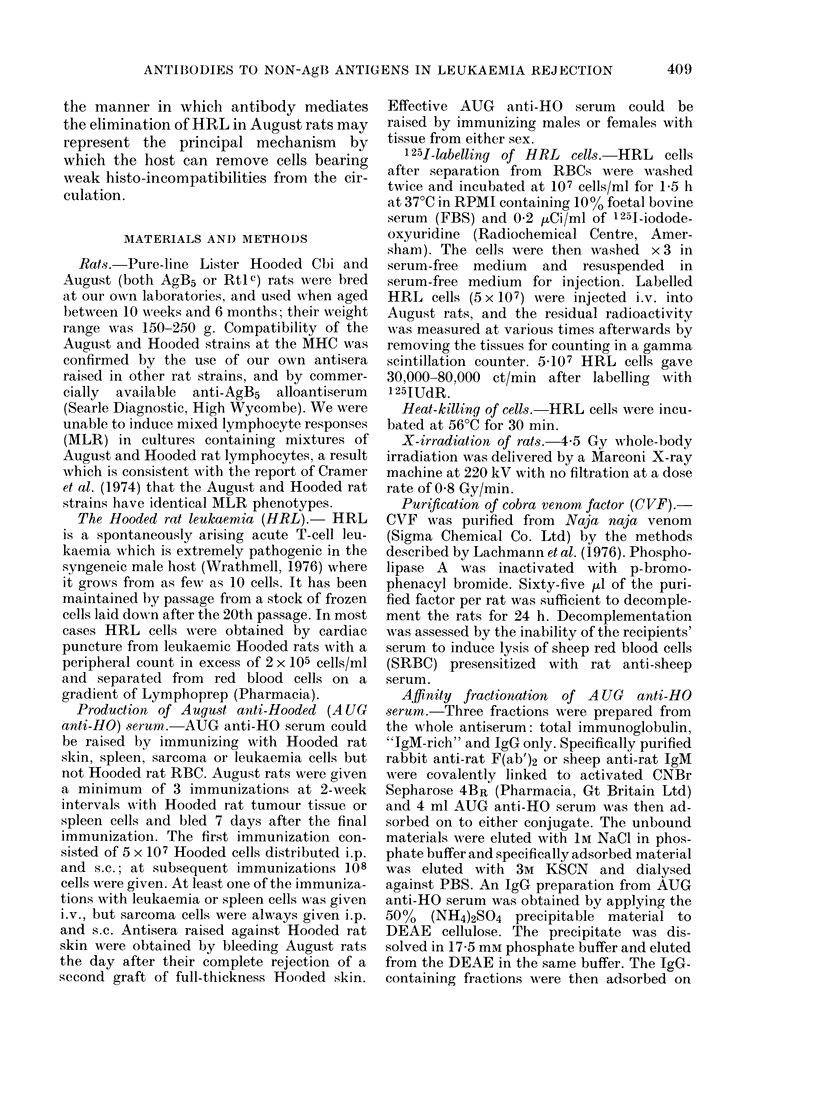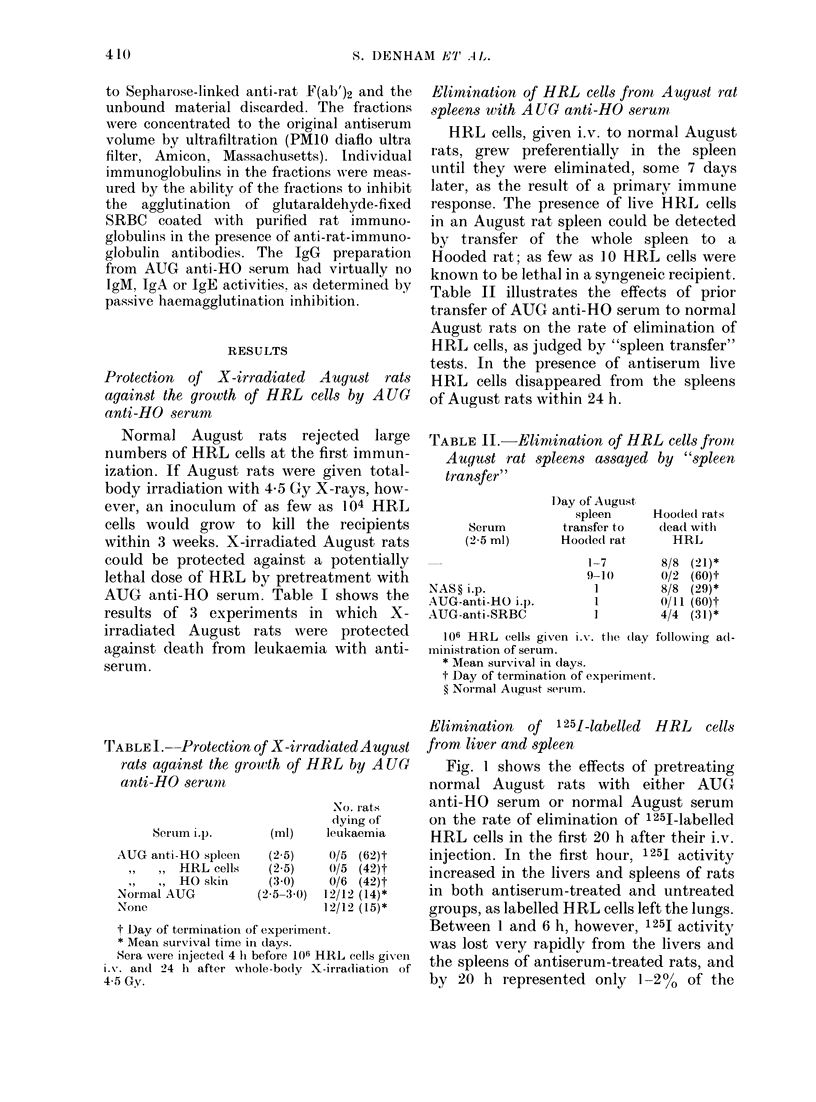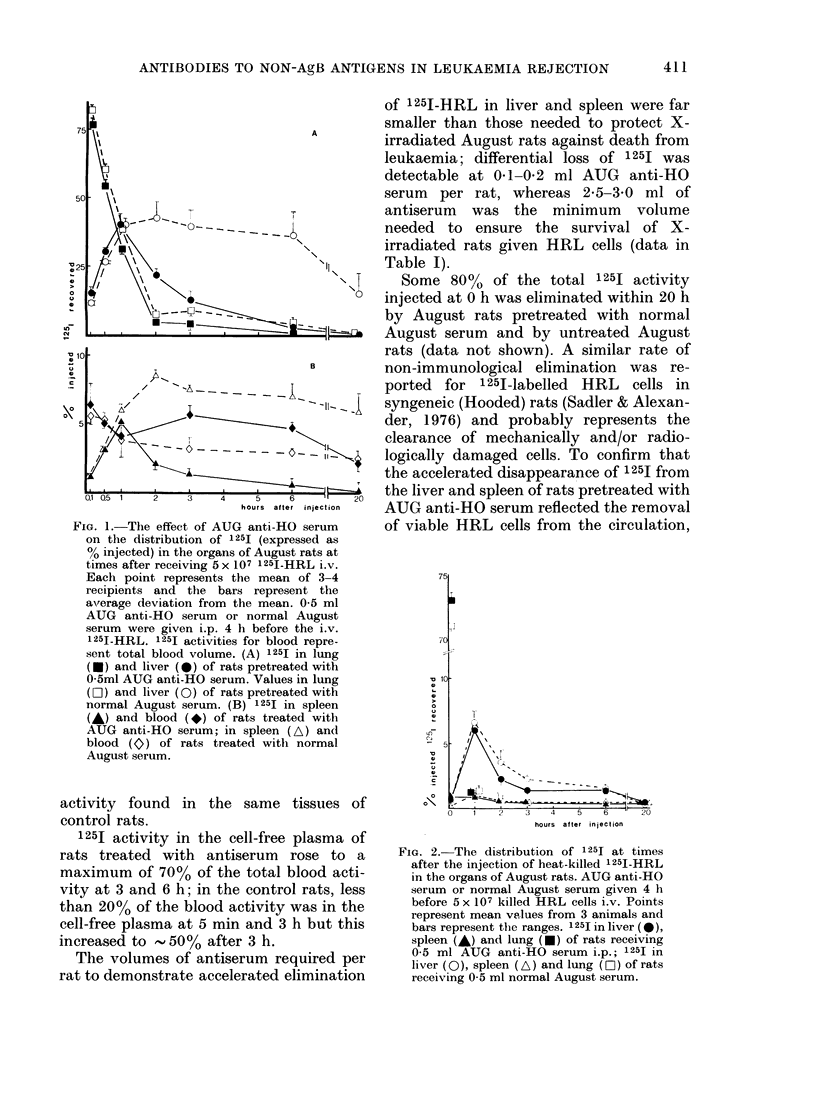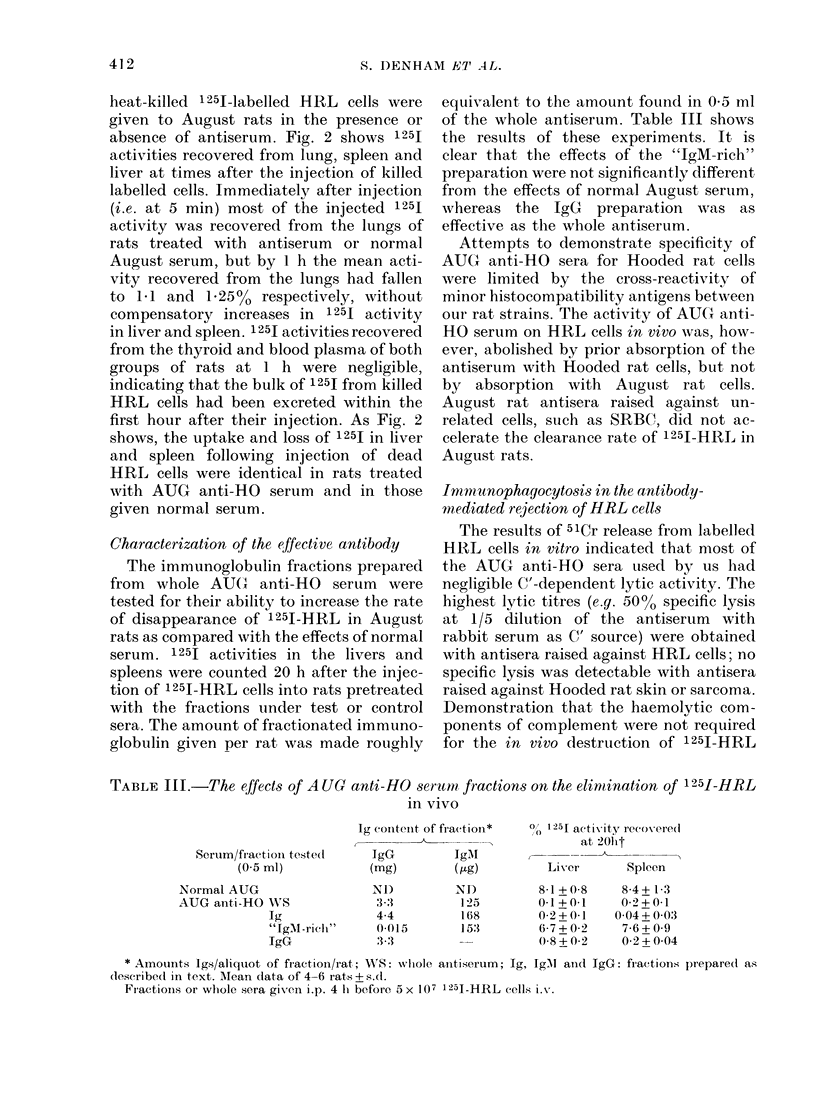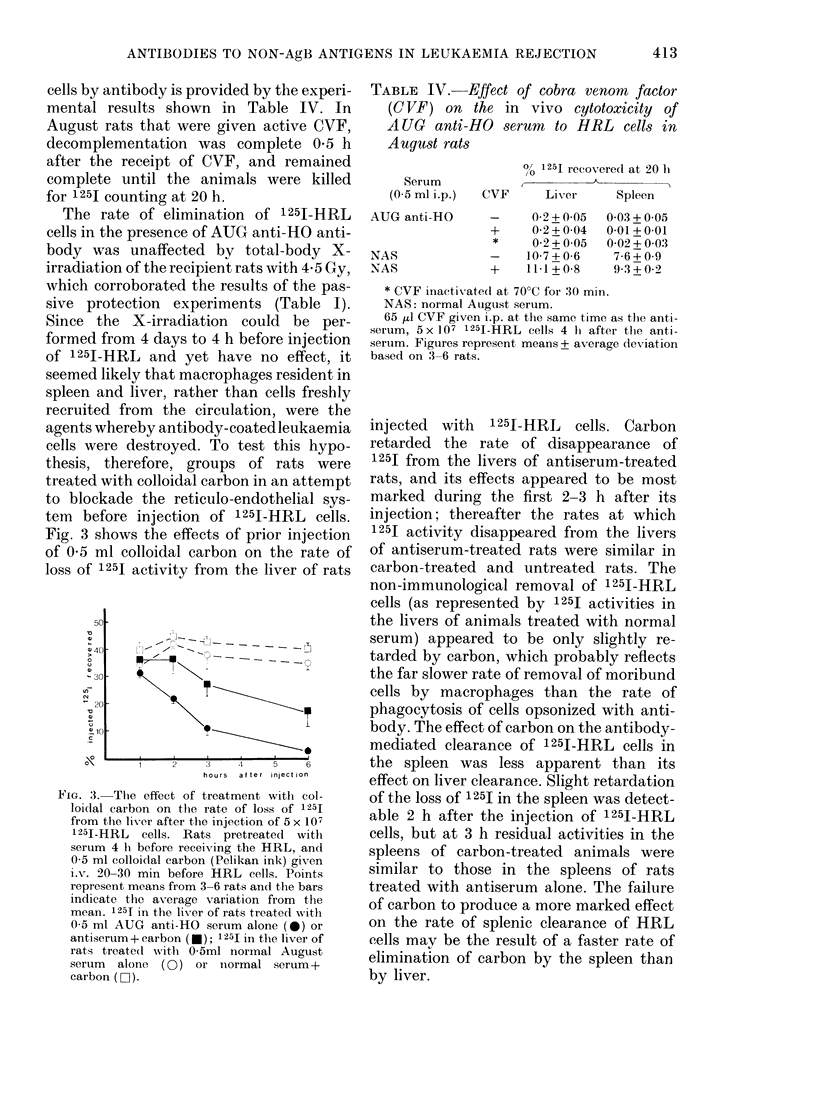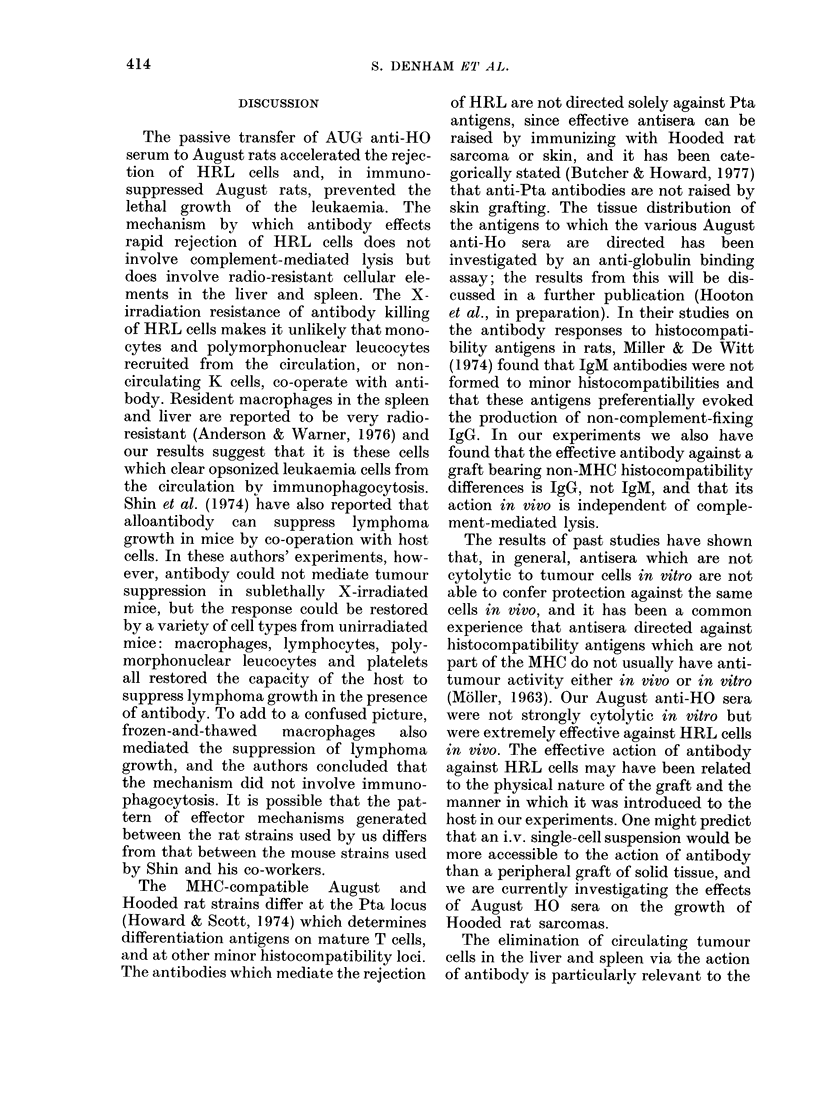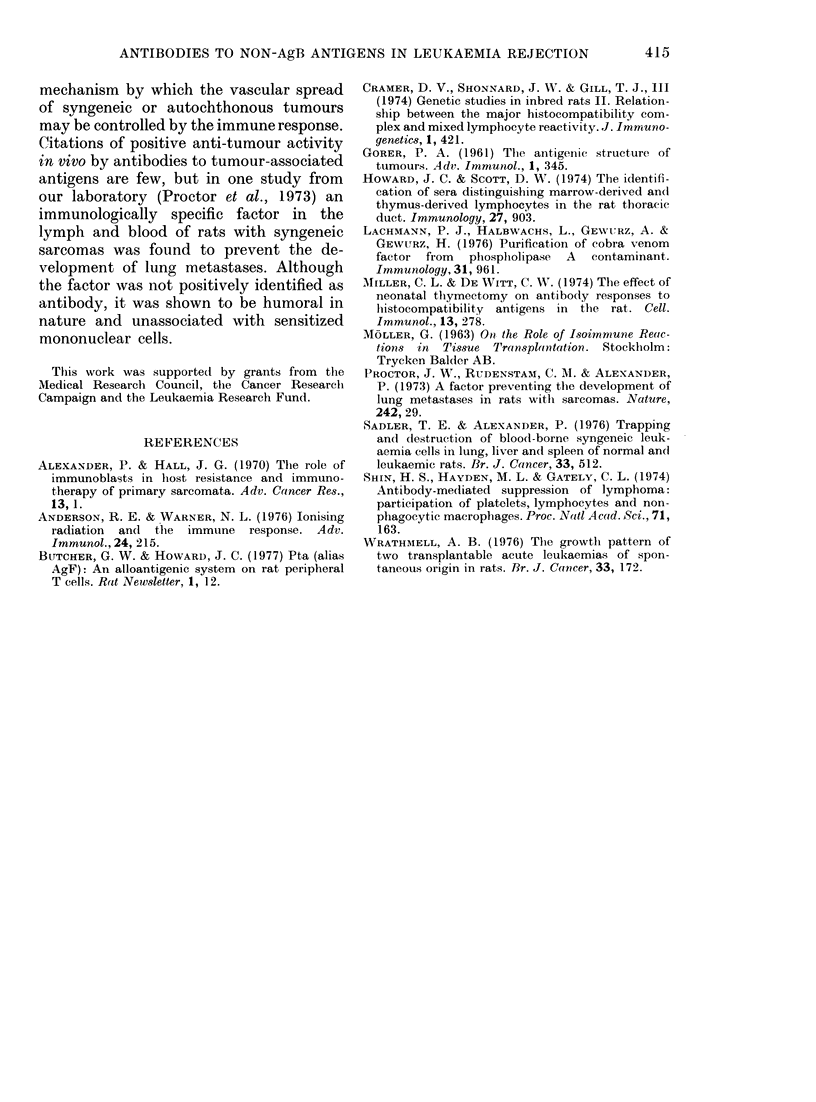# Mechanism by which antibodies to non-AgB antigens mediate rejection of rat leukaemia cells.

**DOI:** 10.1038/bjc.1980.252

**Published:** 1980-09

**Authors:** S. Denham, J. W. Hooton, R. K. Barfoot, P. Alexander, R. Mayol, A. B. Wrathmell

## Abstract

The August and Hooded rat strains are compatible at the major histocompatibility locus (both are AgB5 or Rtlc). Antisera against the minor histocompatibility antigens of Hooded rats were raised by immunizing August rats with grafts of tumours or normal tissue. Such antisera, if transferred to normal unimmunized August rats, cause them to reject i.v. administered Hooded rat leukemia (HRL) cells within a few hours, and X-irradiated August rats, for whom a graft of HRL is lethal, can survive indefinitely if pretreated with the antiserum. The distribution of 125I-labelled HRL cells in the tissues of August rats was followed at times after their injection, and it was found that, in the presence of antiserum, i.v. administered leukaemic cells are rapidly destroyed in the liver and spleen. The active component of the antiserum is IgG antibody, and its action is independent of the lytic elements of complement. Antibody-mediated splenic and hepatic clearance of the leukaemia cells is unaffected by total-body X-irradiation but reduced by treating the rats with colloidal carbon. The data are consistent with the hypothesis that the rejection of HRL across the histocompatibility barrier studied is, in the presence of antibody, effected by immunophagocytosis.


*Br. J. Cancer* (1980) **42**, 408

### MECHANISM BY WHICH ANTIBODIES TO NON-AgB ANTIGENS MEDIATE REJECTION OF RAT LEUKAEMIA CELLS

S. DENHAM, J. W. L. HOOTON, R. K. BARFOOT, P. ALEXANDER, R. MAYOL AND A. B. WRATHMELL

*From the Chester Beatty Research Institute, Institute of Cancer Research, Department of Tumour Immunology, Sutton, Surrey*

Received 19 March 1980 Accepted 12 June 1980

**Summary.—The August and Hooded rat strains are compatible at the major histocompatibility locus (both are $AgB_5$ or $Rtl^c$). Antisera against the minor histocompatibility antigens of Hooded rats were raised by immunizing August rats with grafts of tumours or normal tissue. Such antisera, if transferred to normal unimmunized August rats, cause them to reject i.v. administered Hooded rat leukaemia (HRL) cells within a few hours, and X-irradiated August rats, for whom a graft of HRL is lethal, can survive indefinitely if pretreated with the antiserum. The distribution of $^{125}I$-labelled HRL cells in the tissues of August rats was followed at times after their injection, and it was found that, in the presence of antiserum, i.v. administered leukaemic cells are rapidly destroyed in the liver and spleen. The active component of the antiserum is IgG antibody, and its action is independent of the lytic elements of complement. Antibody-mediated splenic and hepatic clearance of the leukaemia cells is unaffected by total-body X-irradiation but reduced by treating the rats with colloidal carbon. The data are consistent with the hypothesis that the rejection of HRL across the histocompatibility barrier studied is, in the presence of antibody, effected by immunophagocytosis.**

THE ROLE of humoral antibody in the rejection of neoplastic tissue has been at issue for many years. The results of early experiments (see reviews by Gorer, 1961 and Möller, 1963) established that tumour cells were susceptible to the cytolytic action of antibodies and complement *in vitro* and that passively administered cytotoxic antisera would eliminate homografts of leukaemia cells, though not usually solid tumour grafts, *in vivo*. In most of these experiments, however, demonstrably cytotoxic antisera could be raised only by immunizing strains of animals with different major histocompatibility (MHC) antigens from those of the donor of the tumour graft; antisera to non-MHC histocompatibility antigens were not usually cytotoxic *in vitro*, and the passive transfer of such antisera frequently enhanced the growth of leukaemia and solid-tumour grafts (Möller, 1963) rather than their rejection.

In our studies on the immune responses between the MHC-compatible August and Lister Hooded rat strains, we have found that antisera raised in August rats against the minor histo-incompatibilities of Hooded rats may have weak or non-existent complement-mediated cytotoxicity to Hooded rat leukaemia (HRL) cells *in vitro*, and yet effect rapid rejection of the grafted leukaemia cells on passive transfer of the serum *in vivo*. In this paper we demonstrate that August anti-Hooded serum mediates the rapid removal of grafted leukaemia cells from the circulation by their destruction in the spleen and liver. Antibody has been implicated in the control of haematogenous spread of syngeneic and autochthonous tumours (Alexander & Hall, 1970) and we suggest that the manner in which antibody mediates the elimination of HRL in August rats may represent the principal mechanism by which the host can remove cells bearing weak histo-incompatibilities from the circulation.

### MATERIALS AND METHODS

*Rats.*—Pure-line Lister Hooded Cbi and August (both $AgB_5$ or $Rt1^c$) rats were bred at our own laboratories, and used when aged between 10 weeks and 6 months; their weight range was 150–250 g. Compatibility of the August and Hooded strains at the MHC was confirmed by the use of our own antisera raised in other rat strains, and by commercially available anti-$AgB_5$ alloantiserum (Searle Diagnostic, High Wycombe). We were unable to induce mixed lymphocyte responses (MLR) in cultures containing mixtures of August and Hooded rat lymphocytes, a result which is consistent with the report of Cramer *et al.* (1974) that the August and Hooded rat strains have identical MLR phenotypes.

*The Hooded rat leukaemia* (*HRL*).— HRL is a spontaneously arising acute T-cell leukaemia which is extremely pathogenic in the syngeneic male host (Wrathmell, 1976) where it grows from as few as 10 cells. It has been maintained by passage from a stock of frozen cells laid down after the 20th passage. In most cases HRL cells were obtained by cardiac puncture from leukaemic Hooded rats with a peripheral count in excess of $2 \times 10^5$ cells/ml and separated from red blood cells on a gradient of Lymphoprep (Pharmacia).

*Production of August anti-Hooded* (*AUG anti-HO*) *serum.*—AUG anti-HO serum could be raised by immunizing with Hooded rat skin, spleen, sarcoma or leukaemia cells but not Hooded rat RBC. August rats were given a minimum of 3 immunizations at 2-week intervals with Hooded rat tumour tissue or spleen cells and bled 7 days after the final immunization. The first immunization consisted of $5 \times 10^7$ Hooded cells distributed i.p. and s.c.; at subsequent immunizations $10^8$ cells were given. At least one of the immunizations with leukaemia or spleen cells was given i.v., but sarcoma cells were always given i.p. and s.c. Antisera raised against Hooded rat skin were obtained by bleeding August rats the day after their complete rejection of a second graft of full-thickness Hooded skin. Effective AUG anti-HO serum could be raised by immunizing males or females with tissue from either sex.

*$^{125}$I-labelling of HRL cells.*—HRL cells after separation from RBCs were washed twice and incubated at $10^7$ cells/ml for 1·5 h at 37°C in RPMI containing 10% foetal bovine serum (FBS) and 0·2 $\mu$Ci/ml of $^{125}$I-iododeoxyuridine (Radiochemical Centre, Amersham). The cells were then washed $\times 3$ in serum-free medium and resuspended in serum-free medium for injection. Labelled HRL cells ($5 \times 10^7$) were injected i.v. into August rats, and the residual radioactivity was measured at various times afterwards by removing the tissues for counting in a gamma scintillation counter. $5 \cdot 10^7$ HRL cells gave 30,000–80,000 ct/min after labelling with $^{125}$IUdR.

*Heat-killing of cells.*—HRL cells were incubated at 56°C for 30 min.

*X-irradiation of rats.*—4·5 Gy whole-body irradiation was delivered by a Marconi X-ray machine at 220 kV with no filtration at a dose rate of 0·8 Gy/min.

*Purification of cobra venom factor* (*CVF*).—CVF was purified from *Naja naja* venom (Sigma Chemical Co. Ltd) by the methods described by Lachmann *et al.* (1976). Phospholipase A was inactivated with p-bromophenacyl bromide. Sixty-five $\mu$l of the purified factor per rat was sufficient to decomplement the rats for 24 h. Decomplementation was assessed by the inability of the recipients' serum to induce lysis of sheep red blood cells (SRBC) presensitized with rat anti-sheep serum.

*Affinity fractionation of AUG anti-HO serum.*—Three fractions were prepared from the whole antiserum: total immunoglobulin, "IgM-rich" and IgG only. Specifically purified rabbit anti-rat $F(ab')_2$ or sheep anti-rat IgM were covalently linked to activated CNBr Sepharose $4B_R$ (Pharmacia, Gt Britain Ltd) and 4 ml AUG anti-HO serum was then adsorbed on to either conjugate. The unbound materials were eluted with 1M NaCl in phosphate buffer and specifically adsorbed material was eluted with 3M KSCN and dialysed against PBS. An IgG preparation from AUG anti-HO serum was obtained by applying the 50% $(NH_4)_2SO_4$ precipitable material to DEAE cellulose. The precipitate was dissolved in 17·5 mM phosphate buffer and eluted from the DEAE in the same buffer. The IgG-containing fractions were then adsorbed on to Sepharose-linked anti-rat $F(ab')_2$ and the unbound material discarded. The fractions were concentrated to the original antiserum volume by ultrafiltration (PM10 diaflo ultra filter, Amicon, Massachusetts). Individual immunoglobulins in the fractions were measured by the ability of the fractions to inhibit the agglutination of glutaraldehyde-fixed SRBC coated with purified rat immunoglobulins in the presence of anti-rat-immunoglobulin antibodies. The IgG preparation from AUG anti-HO serum had virtually no IgM, IgA or IgE activities, as determined by passive haemagglutination inhibition.

### RESULTS

### *Protection of X-irradiated August rats against the growth of HRL cells by AUG anti-HO serum*

Normal August rats rejected large numbers of HRL cells at the first immunization. If August rats were given total-body irradiation with 4·5 Gy X-rays, however, an inoculum of as few as $10^4$ HRL cells would grow to kill the recipients within 3 weeks. X-irradiated August rats could be protected against a potentially lethal dose of HRL by pretreatment with AUG anti-HO serum. Table I shows the results of 3 experiments in which X-irradiated August rats were protected against death from leukaemia with antiserum.

TABLE I.—*Protection of X-irradiated August rats against the growth of HRL by AUG anti-HO serum*

| Serum i.p. | (ml) | No. rats dying of leukaemia |
|---|---|---|
| AUG anti-HO spleen | (2·5) | 0/5 (62)† |
| ,, ,, HRL cells | (2·5) | 0/5 (42)† |
| ,, ,, HO skin | (3·0) | 0/6 (42)† |
| Normal AUG | (2·5–3·0) | 12/12 (14)* |
| None | | 12/12 (15)* |

† Day of termination of experiment.
* Mean survival time in days.
Sera were injected 4 h before $10^6$ HRL cells given i.v. and 24 h after whole-body X-irradiation of 4·5 Gy.

### *Elimination of HRL cells from August rat spleens with AUG anti-HO serum*

HRL cells, given i.v. to normal August rats, grew preferentially in the spleen until they were eliminated, some 7 days later, as the result of a primary immune response. The presence of live HRL cells in an August rat spleen could be detected by transfer of the whole spleen to a Hooded rat; as few as 10 HRL cells were known to be lethal in a syngeneic recipient. Table II illustrates the effects of prior transfer of AUG anti-HO serum to normal August rats on the rate of elimination of HRL cells, as judged by "spleen transfer" tests. In the presence of antiserum live HRL cells disappeared from the spleens of August rats within 24 h.

TABLE II.—*Elimination of HRL cells from August rat spleens assayed by "spleen transfer"*

| Serum (2·5 ml) | Day of August spleen transfer to Hooded rat | Hooded rats dead with HRL |
|---|---|---|
| — | 1–7 | 8/8 (21)* |
| | 9–10 | 0/2 (60)† |
| NAS§ i.p. | 1 | 8/8 (29)* |
| AUG-anti-HO i.p. | 1 | 0/11 (60)† |
| AUG-anti-SRBC | 1 | 4/4 (31)* |

$10^6$ HRL cells given i.v. the day following administration of serum.

* Mean survival in days.

† Day of termination of experiment.

§ Normal August serum.

### *Elimination of $^{125}$I-labelled HRL cells from liver and spleen*

Fig. 1 shows the effects of pretreating normal August rats with either AUG anti-HO serum or normal August serum on the rate of elimination of $^{125}$I-labelled HRL cells in the first 20 h after their i.v. injection. In the first hour, $^{125}$I activity increased in the livers and spleens of rats in both antiserum-treated and untreated groups, as labelled HRL cells left the lungs. Between 1 and 6 h, however, $^{125}$I activity was lost very rapidly from the livers and the spleens of antiserum-treated rats, and by 20 h represented only 1–2% of the

FIG. 1.—The effect of AUG anti-HO serum on the distribution of $^{125}I$ (expressed as % injected) in the organs of August rats at times after receiving $5 \times 10^7$ $^{125}I$-HRL i.v. Each point represents the mean of 3–4 recipients and the bars represent the average deviation from the mean. 0·5 ml AUG anti-HO serum or normal August serum were given i.p. 4 h before the i.v. $^{125}I$-HRL. $^{125}I$ activities for blood represent total blood volume. (A) $^{125}I$ in lung (■) and liver (●) of rats pretreated with 0·5ml AUG anti-HO serum. Values in lung (□) and liver (○) of rats pretreated with normal August serum. (B) $^{125}I$ in spleen (▲) and blood (◆) of rats treated with AUG anti-HO serum; in spleen (△) and blood (◇) of rats treated with normal August serum.

activity found in the same tissues of control rats.

$^{125}I$ activity in the cell-free plasma of rats treated with antiserum rose to a maximum of 70% of the total blood activity at 3 and 6 h; in the control rats, less than 20% of the blood activity was in the cell-free plasma at 5 min and 3 h but this increased to ~50% after 3 h.

The volumes of antiserum required per rat to demonstrate accelerated elimination of $^{125}I$-HRL in liver and spleen were far smaller than those needed to protect X-irradiated August rats against death from leukaemia; differential loss of $^{125}I$ was detectable at 0·1–0·2 ml AUG anti-HO serum per rat, whereas 2·5–3·0 ml of antiserum was the minimum volume needed to ensure the survival of X-irradiated rats given HRL cells (data in Table I).

Some 80% of the total $^{125}I$ activity injected at 0 h was eliminated within 20 h by August rats pretreated with normal August serum and by untreated August rats (data not shown). A similar rate of non-immunological elimination was reported for $^{125}I$-labelled HRL cells in syngeneic (Hooded) rats (Sadler & Alexander, 1976) and probably represents the clearance of mechanically and/or radiologically damaged cells. To confirm that the accelerated disappearance of $^{125}I$ from the liver and spleen of rats pretreated with AUG anti-HO serum reflected the removal of viable HRL cells from the circulation,

FIG. 2.—The distribution of $^{125}I$ at times after the injection of heat-killed $^{125}I$-HRL in the organs of August rats. AUG anti-HO serum or normal August serum given 4 h before $5 \times 10^7$ killed HRL cells i.v. Points represent mean values from 3 animals and bars represent the ranges. $^{125}I$ in liver (●), spleen (▲) and lung (■) of rats receiving 0·5 ml AUG anti-HO serum i.p.; $^{125}I$ in liver (○), spleen (△) and lung (□) of rats receiving 0·5 ml normal August serum.

heat-killed $^{125}$I-labelled HRL cells were given to August rats in the presence or absence of antiserum. Fig. 2 shows $^{125}$I activities recovered from lung, spleen and liver at times after the injection of killed labelled cells. Immediately after injection (*i.e.* at 5 min) most of the injected $^{125}$I activity was recovered from the lungs of rats treated with antiserum or normal August serum, but by 1 h the mean activity recovered from the lungs had fallen to 1·1 and 1·25% respectively, without compensatory increases in $^{125}$I activity in liver and spleen. $^{125}$I activities recovered from the thyroid and blood plasma of both groups of rats at 1 h were negligible, indicating that the bulk of $^{125}$I from killed HRL cells had been excreted within the first hour after their injection. As Fig. 2 shows, the uptake and loss of $^{125}$I in liver and spleen following injection of dead HRL cells were identical in rats treated with AUG anti-HO serum and in those given normal serum.

*Characterization of the effective antibody*

The immunoglobulin fractions prepared from whole AUG anti-HO serum were tested for their ability to increase the rate of disappearance of $^{125}$I-HRL in August rats as compared with the effects of normal serum. $^{125}$I activities in the livers and spleens were counted 20 h after the injection of $^{125}$I-HRL cells into rats pretreated with the fractions under test or control sera. The amount of fractionated immunoglobulin given per rat was made roughly equivalent to the amount found in 0·5 ml of the whole antiserum. Table III shows the results of these experiments. It is clear that the effects of the "IgM-rich" preparation were not significantly different from the effects of normal August serum, whereas the IgG preparation was as effective as the whole antiserum.

Attempts to demonstrate specificity of AUG anti-HO sera for Hooded rat cells were limited by the cross-reactivity of minor histocompatibility antigens between our rat strains. The activity of AUG anti-HO serum on HRL cells *in vivo* was, however, abolished by prior absorption of the antiserum with Hooded rat cells, but not by absorption with August rat cells. August rat antisera raised against unrelated cells, such as SRBC, did not accelerate the clearance rate of $^{125}$I-HRL in August rats.

*Immunophagocytosis in the antibody-mediated rejection of HRL cells*

The results of $^{51}$Cr release from labelled HRL cells *in vitro* indicated that most of the AUG anti-HO sera used by us had negligible C′-dependent lytic activity. The highest lytic titres (*e.g.* 50% specific lysis at 1/5 dilution of the antiserum with rabbit serum as C′ source) were obtained with antisera raised against HRL cells; no specific lysis was detectable with antisera raised against Hooded rat skin or sarcoma. Demonstration that the haemolytic components of complement were not required for the *in vivo* destruction of $^{125}$I-HRL

TABLE III.—*The effects of AUG anti-HO serum fractions on the elimination of $^{125}$I-HRL* in vivo

| Serum/fraction tested (0·5 ml) | | Ig content of fraction* IgG (mg) | IgM (μg) | % $^{125}$I activity recovered at 20h† Liver | Spleen |
|---|---|---|---|---|---|
| Normal AUG | | ND | ND | 8·1 ± 0·8 | 8·4 ± 1·3 |
| AUG anti-HO | WS | 3·3 | 125 | 0·1 ± 0·1 | 0·2 ± 0·1 |
| | Ig | 4·4 | 168 | 0·2 ± 0·1 | 0·04 ± 0·03 |
| | "IgM-rich" | 0·015 | 153 | 6·7 ± 0·2 | 7·6 ± 0·9 |
| | IgG | 3·3 | — | 0·8 ± 0·2 | 0·2 ± 0·04 |

* Amounts Igs/aliquot of fraction/rat; WS: whole antiserum; Ig, IgM and IgG: fractions prepared as described in text. Mean data of 4–6 rats ± s.d.

Fractions or whole sera given i.p. 4 h before 5 × 10$^{7}$ $^{125}$I-HRL cells i.v.

cells by antibody is provided by the experimental results shown in Table IV. In August rats that were given active CVF, decomplementation was complete 0·5 h after the receipt of CVF, and remained complete until the animals were killed for $^{125}$I counting at 20 h.

The rate of elimination of $^{125}$I-HRL cells in the presence of AUG anti-HO antibody was unaffected by total-body X-irradiation of the recipient rats with 4·5 Gy, which corroborated the results of the passive protection experiments (Table I). Since the X-irradiation could be performed from 4 days to 4 h before injection of $^{125}$I-HRL and yet have no effect, it seemed likely that macrophages resident in spleen and liver, rather than cells freshly recruited from the circulation, were the agents whereby antibody-coated leukaemia cells were destroyed. To test this hypothesis, therefore, groups of rats were treated with colloidal carbon in an attempt to blockade the reticulo-endothelial system before injection of $^{125}$I-HRL cells. Fig. 3 shows the effects of prior injection of 0·5 ml colloidal carbon on the rate of loss of $^{125}$I activity from the liver of rats

FIG. 3.—The effect of treatment with colloidal carbon on the rate of loss of $^{125}$I from the liver after the injection of $5 \times 10^7$ $^{125}$I-HRL cells. Rats pretreated with serum 4 h before receiving the HRL, and 0·5 ml colloidal carbon (Pelikan ink) given i.v. 20–30 min before HRL cells. Points represent means from 3–6 rats and the bars indicate the average variation from the mean. $^{125}$I in the liver of rats treated with 0·5 ml AUG anti-HO serum alone (●) or antiserum + carbon (■); $^{125}$I in the liver of rats treated with 0·5ml normal August serum alone (○) or normal serum + carbon (□).

TABLE IV.—*Effect of cobra venom factor (CVF) on the* in vivo *cytotoxicity of AUG anti-HO serum to HRL cells in August rats*

| Serum (0·5 ml i.p.) | CVF | % $^{125}$I recovered at 20 h: Liver | Spleen |
|---|---|---|---|
| AUG anti-HO | – | 0·2 ± 0·05 | 0·03 ± 0·05 |
| | + | 0·2 ± 0·04 | 0·01 ± 0·01 |
| | * | 0·2 ± 0·05 | 0·02 ± 0·03 |
| NAS | – | 10·7 ± 0·6 | 7·6 ± 0·9 |
| NAS | + | 11·1 ± 0·8 | 9·3 ± 0·2 |

* CVF inactivated at 70°C for 30 min.
NAS: normal August serum.
65 μl CVF given i.p. at the same time as the antiserum, $5 \times 10^7$ $^{125}$I-HRL cells 4 h after the antiserum. Figures represent means ± average deviation based on 3–6 rats.

injected with $^{125}$I-HRL cells. Carbon retarded the rate of disappearance of $^{125}$I from the livers of antiserum-treated rats, and its effects appeared to be most marked during the first 2–3 h after its injection; thereafter the rates at which $^{125}$I activity disappeared from the livers of antiserum-treated rats were similar in carbon-treated and untreated rats. The non-immunological removal of $^{125}$I-HRL cells (as represented by $^{125}$I activities in the livers of animals treated with normal serum) appeared to be only slightly retarded by carbon, which probably reflects the far slower rate of removal of moribund cells by macrophages than the rate of phagocytosis of cells opsonized with antibody. The effect of carbon on the antibody-mediated clearance of $^{125}$I-HRL cells in the spleen was less apparent than its effect on liver clearance. Slight retardation of the loss of $^{125}$I in the spleen was detectable 2 h after the injection of $^{125}$I-HRL cells, but at 3 h residual activities in the spleens of carbon-treated animals were similar to those in the spleens of rats treated with antiserum alone. The failure of carbon to produce a more marked effect on the rate of splenic clearance of HRL cells may be the result of a faster rate of elimination of carbon by the spleen than by liver.

### DISCUSSION

The passive transfer of AUG anti-HO serum to August rats accelerated the rejection of HRL cells and, in immunosuppressed August rats, prevented the lethal growth of the leukaemia. The mechanism by which antibody effects rapid rejection of HRL cells does not involve complement-mediated lysis but does involve radio-resistant cellular elements in the liver and spleen. The X-irradiation resistance of antibody killing of HRL cells makes it unlikely that monocytes and polymorphonuclear leucocytes recruited from the circulation, or non-circulating K cells, co-operate with antibody. Resident macrophages in the spleen and liver are reported to be very radio-resistant (Anderson & Warner, 1976) and our results suggest that it is these cells which clear opsonized leukaemia cells from the circulation by immunophagocytosis. Shin *et al.* (1974) have also reported that alloantibody can suppress lymphoma growth in mice by co-operation with host cells. In these authors' experiments, however, antibody could not mediate tumour suppression in sublethally X-irradiated mice, but the response could be restored by a variety of cell types from unirradiated mice: macrophages, lymphocytes, polymorphonuclear leucocytes and platelets all restored the capacity of the host to suppress lymphoma growth in the presence of antibody. To add to a confused picture, frozen-and-thawed macrophages also mediated the suppression of lymphoma growth, and the authors concluded that the mechanism did not involve immunophagocytosis. It is possible that the pattern of effector mechanisms generated between the rat strains used by us differs from that between the mouse strains used by Shin and his co-workers.

The MHC-compatible August and Hooded rat strains differ at the Pta locus (Howard & Scott, 1974) which determines differentiation antigens on mature T cells, and at other minor histocompatibility loci. The antibodies which mediate the rejection of HRL are not directed solely against Pta antigens, since effective antisera can be raised by immunizing with Hooded rat sarcoma or skin, and it has been categorically stated (Butcher & Howard, 1977) that anti-Pta antibodies are not raised by skin grafting. The tissue distribution of the antigens to which the various August anti-Ho sera are directed has been investigated by an anti-globulin binding assay; the results from this will be discussed in a further publication (Hooton *et al.*, in preparation). In their studies on the antibody responses to histocompatibility antigens in rats, Miller & De Witt (1974) found that IgM antibodies were not formed to minor histocompatibilities and that these antigens preferentially evoked the production of non-complement-fixing IgG. In our experiments we also have found that the effective antibody against a graft bearing non-MHC histocompatibility differences is IgG, not IgM, and that its action *in vivo* is independent of complement-mediated lysis.

The results of past studies have shown that, in general, antisera which are not cytolytic to tumour cells *in vitro* are not able to confer protection against the same cells *in vivo*, and it has been a common experience that antisera directed against histocompatibility antigens which are not part of the MHC do not usually have anti-tumour activity either *in vivo* or *in vitro* (Möller, 1963). Our August anti-HO sera were not strongly cytolytic *in vitro* but were extremely effective against HRL cells *in vivo*. The effective action of antibody against HRL cells may have been related to the physical nature of the graft and the manner in which it was introduced to the host in our experiments. One might predict that an i.v. single-cell suspension would be more accessible to the action of antibody than a peripheral graft of solid tissue, and we are currently investigating the effects of August HO sera on the growth of Hooded rat sarcomas.

The elimination of circulating tumour cells in the liver and spleen via the action of antibody is particularly relevant to the mechanism by which the vascular spread of syngeneic or autochthonous tumours may be controlled by the immune response. Citations of positive anti-tumour activity *in vivo* by antibodies to tumour-associated antigens are few, but in one study from our laboratory (Proctor *et al.*, 1973) an immunologically specific factor in the lymph and blood of rats with syngeneic sarcomas was found to prevent the development of lung metastases. Although the factor was not positively identified as antibody, it was shown to be humoral in nature and unassociated with sensitized mononuclear cells.

This work was supported by grants from the Medical Research Council, the Cancer Research Campaign and the Leukaemia Research Fund.

### REFERENCES

ALEXANDER, P. & HALL, J. G. (1970) The role of immunoblasts in host resistance and immunotherapy of primary sarcomata. *Adv. Cancer Res.*, **13,** 1.

ANDERSON, R. E. & WARNER, N. L. (1976) Ionising radiation and the immune response. *Adv. Immunol.*, **24,** 215.

BUTCHER, G. W. & HOWARD, J. C. (1977) Pta (alias AgF): An alloantigenic system on rat peripheral T cells. *Rat Newsletter*, **1,** 12.

CRAMER, D. V., SHONNARD, J. W. & GILL, T. J., III (1974) Genetic studies in inbred rats II. Relationship between the major histocompatibility complex and mixed lymphocyte reactivity. *J. Immunogenetics*, **1,** 421.

GORER, P. A. (1961) The antigenic structure of tumours. *Adv. Immunol.*, **1,** 345.

HOWARD, J. C. & SCOTT, D. W. (1974) The identification of sera distinguishing marrow-derived and thymus-derived lymphocytes in the rat thoracic duct. *Immunology*, **27,** 903.

LACHMANN, P. J., HALBWACHS, L., GEWURZ, A. & GEWURZ, H. (1976) Purification of cobra venom factor from phospholipase A contaminant. *Immunology*, **31,** 961.

MILLER, C. L. & DE WITT, C. W. (1974) The effect of neonatal thymectomy on antibody responses to histocompatibility antigens in the rat. *Cell. Immunol.*, **13,** 278.

MÖLLER, G. (1963) *On the Role of Isoimmune Reactions in Tissue Transplantation.* Stockholm: Trycken Balder AB.

PROCTOR, J. W., RUDENSTAM, C. M. & ALEXANDER, P. (1973) A factor preventing the development of lung metastases in rats with sarcomas. *Nature*, **242,** 29.

SADLER, T. E. & ALEXANDER, P. (1976) Trapping and destruction of blood-borne syngeneic leukaemia cells in lung, liver and spleen of normal and leukaemic rats. *Br. J. Cancer*, **33,** 512.

SHIN, H. S., HAYDEN, M. L. & GATELY, C. L. (1974) Antibody-mediated suppression of lymphoma: participation of platelets, lymphocytes and nonphagocytic macrophages. *Proc. Natl Acad. Sci.*, **71,** 163.

WRATHMELL, A. B. (1976) The growth pattern of two transplantable acute leukaemias of spontaneous origin in rats. *Br. J. Cancer*, **33,** 172.